# Adjuvant Chemotherapy in Uterine Leiomyosarcoma: Trends and Factors Impacting Usage

**DOI:** 10.1155/2019/3561501

**Published:** 2019-02-10

**Authors:** Dhara Patel, Elizabeth Handorf, Margaret von Mehren, Lainie Martin, Sujana Movva

**Affiliations:** ^1^Northeast Ohio Medical University, Rootstown, OH 44272, USA; ^2^Department of Biostatistics, Fox Chase Cancer Center, Philadelphia, PA 19111, USA; ^3^Department of Hematology and Oncology, Fox Chase Cancer Center, Philadelphia, PA 19111, USA

## Abstract

**Objectives:**

The benefit of adjuvant chemotherapy in patients with localized uterine leiomyosarcoma (LMS) remains unclear due to a lack of randomized studies and data only from small retrospective series to rely on. We sought to identify factors associated with the administration of chemotherapy and to determine the trends in the usage of adjuvant chemotherapy in patients with nonmetastatic uterine LMS.

**Methods:**

Patients diagnosed with nonmetastatic uterine LMS between 2004 and 2014 were identified from the National Cancer Database (NCDB). Multiple regression was used to determine factors with a significant impact on patient receipt of chemotherapy. Kaplan–Meier curves and the Cox model were used to determine the effect of adjuvant chemotherapy on overall survival (OS).

**Results:**

2,732 uterine LMS patients were identified. Patients older than 65 were less likely to receive chemotherapy than their younger counterparts. Patients with stage I or stage II cancer were less likely to receive chemotherapy, whereas individuals with positive regional lymph nodes and those who had received radiation were more likely. In this cohort, adjuvant chemotherapy had no significant impact on OS (HR, 1.04; 95% CI, 0.90–1.22; *P*=0.5768). However, administration of chemotherapy significantly increased from 2004 to 2014 (*P* < 0.0001).

**Conclusions:**

Expected tumor characteristics such as higher stage of tumor were associated with receipt of chemotherapy. Although adjuvant chemotherapy demonstrated no benefit over observation on OS in patients with nonmetastatic LMS, the number of patients being treated with chemotherapy continued to increase from 2004 to 2014.

## 1. Introduction

Soft tissue sarcomas (STSs) are a rare group of mesenchymal tumors that account for approximately 1% of adult cancers in the US. These tumors are highly anatomically, histologically, and biologically heterogeneous, making treatment and prognostication complex. Leiomyosarcoma (LMS) is one of the more common types of STS. Uterine LMS is a rare disease, which makes its study and corresponding development of treatment guidelines difficult. For localized uterine LMS, surgery, such as hysterectomy, is the primary modality of therapy. Physicians may also recommend bilateral salpingo-oophorectomy (BSO) [1] due to the concern that estrogen and progesterone may drive the risk of recurrence [2].

There are a variety of adjuvant therapies that can be offered for uterine LMS, including radiation, hormone therapy, and chemotherapy. Pelvic radiotherapy is not a common treatment for uterine LMS patients as it has not been shown to significantly improve survival [3, 4]. There have been no prospective studies on the impact of hormone therapy in the adjuvant setting for uterine LMS patients. However, because a significant percentage of uterine LMS expresses estrogen and/or progesterone receptors (ER and/or PR), hormonal blockade is used empirically by some clinicians [5]. Recently, postoperative chemotherapy regimens have been tested with the hope of preventing recurrence and increasing OS. However, due to the rarity of uterine LMS, no conclusive evidence has been found. In 2013, a phase II trial by Hensley et al. showed that adjuvant therapy of fixed-dose-rate gemcitabine-docetaxel followed by doxorubicin in high-grade uterine LMS patients with stage I, II, and IIIA disease resulted in higher than expected progression-free survival (PFS) rates [6]. Unfortunately, the subsequent phase III trial was prematurely closed due to slow accrual. Analysis of the limited dataset suggested that observation should remain the standard of care in uterine LMS [7].

Despite lack of clear benefit, the usage of adjuvant chemotherapy in nonmetastatic uterine leiomyosarcoma has increased significantly in the last decade [8]. However, there have been minimal data on the impact of tumor and demographic factors on chemotherapy receipt. We analyzed the National Cancer Database (NCDB) from 2004 to 2014 to further understand the effects of adjuvant chemotherapy on nonmetastatic uterine LMS and to identify factors involved in the usage of chemotherapy in this disease.

## 2. Materials and Methods

### 2.1. Study Population

Institutional review board approval was not required per the standard operating procedures at our center for studies involving review of deidentified data from a national database. Individuals diagnosed with uterine LMS between January 1, 2004, and December 31, 2014, were identified from the NCDB. This joint organization between the Commission of Cancer of the American College of Surgeons and the American Cancer Society is used by more than 1,500 Commission-accredited cancer programs across the United States to report newly diagnosed cancer cases and oncology outcomes [9].

**Figure 1:**
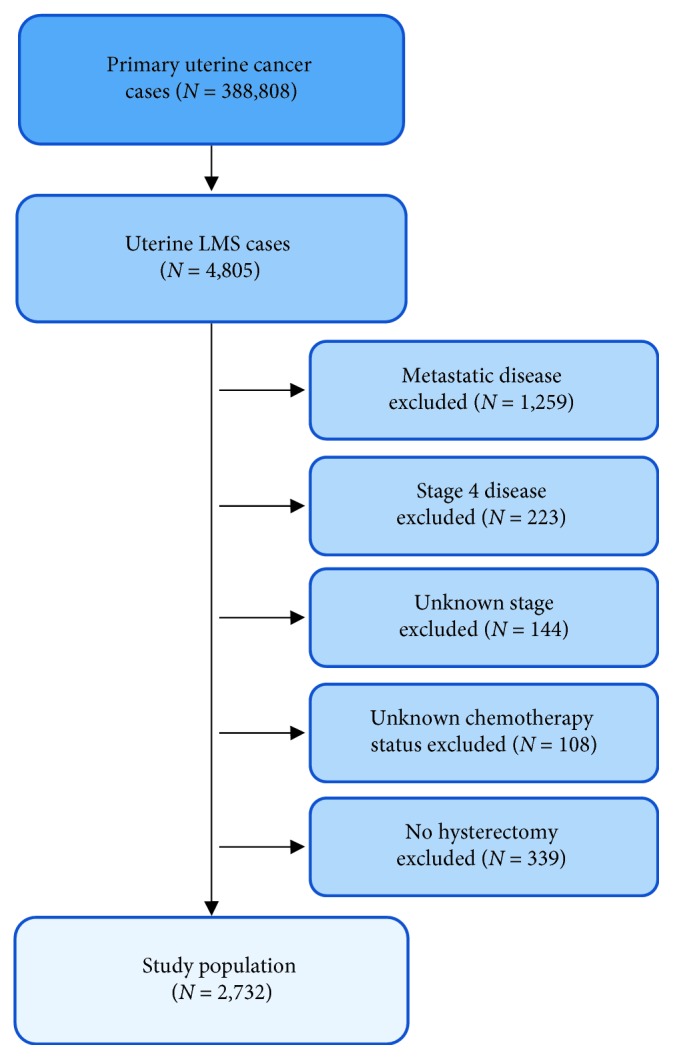
Consort flow diagram used for inclusion and exclusion of patients in this study.

From 2004 to 2014, there were 388,808 cases of primary uterine cancer. Of those, 4,805 had a histologic diagnosis of LMS. Cases with metastatic disease (AJCC stage IV, CS group 4, M1) (*N*=1,259), stage 4 disease (*N*=223), unknown stage disease at presentation (*N*=144), unknown chemotherapy status (*N*=108), and a lack of hysterectomy (*N*=339) were excluded from this study. A total of 2,732 cases were included in this analysis (Figure 1).

### 2.2. Definition of Variables

#### 2.2.1. Tumor Characteristics

Only cases of LMS were included in this study. Cases in the NCDB file were staged either using AJCC or Collaborative Staging (CS). For this study, the stages were defined as I: confined to uterus, II: involving cervical stroma, and III: local or regional spread. The existence of positive regional lymph nodes was also classified.

#### 2.2.2. Patient, Demographic, and Facility Characteristics

Patients' ages at diagnosis were categorized as 18–39, 40–49, 50–64, and ≥65. The race was identified as white, African American, and other. The number of comorbidities was available utilizing the Charlson/Deyo score, where “0” indicates no comorbid conditions. Both median income and education level were determined by linking the patient's zip code with the 2000 US census data. Insurance status was recorded at time of primary diagnosis or treatment. The geographical location of the patient was defined as small and large metropolitan, suburban, and rural. Institutions were categorized by volume based on quartiles with a minimum of 1 case per dataset (0.09 cases/year).

#### 2.2.3. Treatment and Survival

Treatment was documented in the NCDB as administration or lack of administration of adjuvant chemotherapy, radiation therapy, hormone therapy, and oophorectomy. The year of diagnosis was collapsed to 2004–2009 and 2010–2014. Patients may have received single-agent or multiagent chemotherapy, but specific drug regimens are unknown. OS time was defined as months from diagnosis until death or last follow-up. Patients who remained alive at the end of follow-up were considered censored.

### 2.3. Statistical Analysis

We tested the association of receipt of chemotherapy and patient characteristics using chi-squared tests and Cochran–Armitage tests for trend. We then used multivariable logistic regression to simultaneously evaluate predictors of chemotherapy, clustering for within-hospital effects using robust standard errors estimated via generalized estimating equations [10]. Survival time was defined from the date of diagnosis until death or loss to follow-up. Survival was evaluated graphically using nonparametric Kaplan–Meier curves and compared using the log-rank test. A Cox proportional hazard model was fit to estimate the effect of chemotherapy on survival, controlling for known patient factors, using robust standard errors to control for within-hospital effects [11]. We assessed nonproportionality using complementary log-log survival plots and by including an interaction between the treatment and log-time. We also conducted a matched analysis, exact matching on age group and stage, and used propensity score-based matching to balance the remaining covariates [12]. Matching was conducted without replacement within propensity-score calipers. Covariate balance was assessed using standardized differences, where differences of ≤0.1 were considered acceptable [13]. Using the matched sample, we created Kaplan–Meier survival curves and tested for differences using the log-rank test, stratified by matched pair.

## 3. Results

### 3.1. Population Characteristics and Chemotherapy Status

A total of 2,732 patients with uterine LMS meeting eligibility criteria were included in the analysis. Of those, 936 patients received adjuvant chemotherapy, 2,189 underwent an oophorectomy, 598 received radiation, and only 28 patients received adjuvant hormone therapy. Median age in the group receiving chemotherapy was 52 versus 54 in the cohort that did not receive chemotherapy. The majority of patients who were treated with chemotherapy received multiagent regimens (86.9%, *N*=813). Less than 0.5% of patients received chemotherapy neoadjuvantly. In addition, most patients were of white race (74.2%) and had a stage I tumor (81.1%) as per our definition.

On multivariate analysis, the administration of chemotherapy significantly increased from 2004 to 2014 (odds ratio (OR) 1.16 per year; *P* < 0.0001). Patients older than 65 (OR 0.60; *P*=0.0264) and those with Medicare insurance (OR 0.59; *P*=0.0030) were less likely to receive chemotherapy treatment compared to younger patients and those patients who had private insurance. Finally, patients with positive regional lymph nodes (OR 3.83; *P*=0.0004) and those who had received radiation (OR 1.45; *P*=0.0006) were more likely to receive chemotherapy, whereas those with stage I or II uterine LMS were less likely to be treated with chemotherapy (stage I (OR 0.32; *P* < 0.0001); stage II (OR 0.38; *P* < 0.0001)). Though hospital volume appeared to impact chemotherapy receipt on univariate analysis (Table 1), statistical significance was not maintained in the multivariable model. Race, location of patient, comorbidities, hormone therapy, and oophorectomy were not significantly associated with receipt of chemotherapy (Table 2). The impact of socioeconomic status on the administration of chemotherapy remains inconclusive due to seemingly contradictory results of chemotherapy receipt based on education and income levels (Table 2).

### 3.2. Survival Analysis

**Figure 2:**
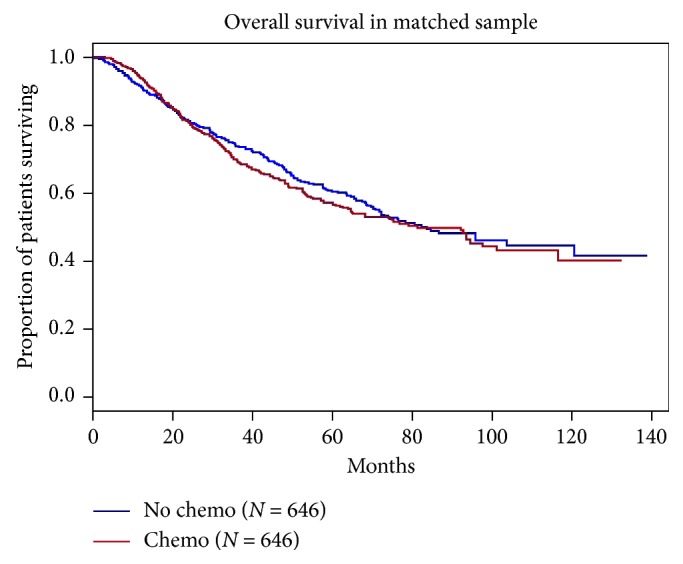
Kaplan–Meier curves of OS based on receipt of chemotherapy. The *x*-axis indicates the survival in months from the date of diagnosis until death or loss to follow-up. The *y*-axis indicates the proportion of patients surviving.

Survival data were available for 2,418 patients, of whom 33% received chemotherapy. Multivariable analysis demonstrated a higher risk of death in patients older than 49 (ages 50–64 (hazard ratio (HR) 2.15; 95% confidence interval (95% CI), 1.51–3.05; *P* < 0.0001); ages 65+ (HR, 3.16; 95% CI, 2.14–4.67; *P* < 0.0001)), African American patients (HR, 1.26; 95% CI, 1.05–1.51; *P*=0.0113), and those with no insurance (HR, 1.40; 95% CI, 1.05–1.88; *P*=0.0243). Individuals who had positive regional lymph nodes (HR, 2.12; 95%CI, 1.29–3.50; *P*=0.0031) were also more likely to die. In contrast, patients with a stage I tumor (HR 0.52; 95% CI 0.42–0.63; *P* < 0.0001) were less likely to die than those with more advanced disease (stage 3). Adjuvant chemotherapy had no impact on OS in patients with nonmetastatic uterine LMS on adjusted analysis (HR, 1.04; 95% CI, 0.90–1.22; *P*=0.5768); however, the survival curves crossed and we found violations of the proportional hazards assumption. We therefore confirmed these results using nonparametric Kaplan–Meier curves, controlling for sociodemographic and tumor factors using propensity score-matching. Matches were identified for 646 of the 808 patients who received chemotherapy and had survival data available, and after matching, all covariates were balanced across the treatment group (*d* ≤ 0.1). Median OS in the matched sample was 82.5 months in the nonchemotherapy group and 81.6 months in the chemotherapy group, and the stratified log-rank test showed no significant difference between groups (*P*=0.80) (Figure 2). Comorbidities, other adjuvant treatments (hormone therapy, radiation therapy), facility volume, and socioeconomic characteristics such as education level and median household income were controlled in all multivariable analyses but were not significantly associated with OS. On further testing, there was no interaction effect between chemotherapy and stage (*P*=0.6528).

## 4. Discussion

Our analysis of the NCDB dataset from 2004 to 2014 suggests that adjuvant chemotherapy has no impact on OS in patients with nonmetastatic uterine LMS. Despite chemotherapy having no proven significant survival benefit over observation, the data collected by the NCDB indicate that 30.5%, 35.6%, and 58.5% of stage I, II, and III uterine LMS patients, respectively, received chemotherapy between the years of 2004–2014. This trend has increased throughout the years. In 2004, only 14.3% of nonmetastatic uterine LMS patients received chemotherapy, whereas the rate climbed to 40.8% in 2014. A retrospective study by Littell et al. which included 111 women with stage I uterine LMS identified from the Kaiser Permanente regional cancer registries showed similar results [8]. They found that the use of adjuvant chemotherapy in stage I uterine LMS significantly increased from 6.5% in 2006–2008 to 46.9% in 2009–2013 (*P* < 0.001). The activity of regimens such as gemcitabine and docetaxel in advanced uterine LMS and phase II studies suggesting a possible benefit for adjuvant chemotherapy may be responsible for this trend [6, 14, 15].

Patients with perceived low-risk disease (stage I or II tumors and without locoregional spread) were significantly less likely to receive chemotherapy. This was also seen in a retrospective study in which, of the uterine LMS patients who did not receive chemotherapy treatment, 68% had a stage I or II tumor [16]. Older patients and those with Medicare were less likely to receive chemotherapy, suggesting that patient selection plays a large role in the administration of adjuvant treatments. Paradoxically, comorbidities had no impact on receipt of chemotherapy. Less data exist in the literature in comparison to our findings. On univariate analysis alone, a multisite, retrospective study of 108 stage I or II high-grade uterine LMS patients diagnosed between 1990 and 2010 demonstrated that other than race/ethnicity, no other demographic characteristics such as mean age, body mass index, comorbidities, mean tumor size, lymph node sampling, and stage of disease had any impact on receipt of chemotherapy [17]. Similar results were found by Littell et al. where race/ethnicity affected chemotherapy receipt, but factors such as median age, body mass index, uterine weight, mitotic index, menopausal status, comorbidities, hysterectomy status, AJCC substage, tumor fragmentation, power morcellation, and adjuvant pelvic radiation did not [8]. Reassuringly, in our analysis, race had no impact on chemotherapy administration. In the analysis by Littell et al., a significantly lower proportion of Hispanic and Asian/Pacific Islanders received chemotherapy when compared to their white counterparts. This was postulated to be due to cultural and/or linguistic factors [8]. Due to small numbers, Asians and Pacific Islanders were grouped together in our analysis with other races and unknown race, but there did not appear to be a difference in receipt of chemotherapy when compared with white women (*P*=0.5965). Patients receiving radiation were also more likely to receive chemotherapy, possibly due to higher stage disease or the use of concomitant chemoradiation.

The five-year survival estimates of uterine LMS provided by the Federation of Gynecology and Obstetrics (FIGO) range from 45 to 76% for stage III to stage I disease, respectively [18]. Accordingly, we found that stage I disease was associated with an improved survival, whereas positive regional lymph nodes were indicative of increased chance of death. Of note, in accordance with standard of care procedures, the majority of patients in our series did not undergo lymph node sampling; therefore, the significance of this result is unclear. We also found that African Americans and older patients had a high risk of death. These results were replicated in a retrospective SEER database study by Kapp et al. which included 1,396 uterine LMS patients diagnosed from 1988 to 2003 [19]. This study demonstrated that older individuals (HR, 1.022; *P* < 0.001), those of African American race (HR, 1.451; *P*=0.011), and those with higher stage disease (HR 1.584; *P* < 0.001) had decreased survival [19]. Limited data exist on the impact of insurance status on survival in uterine LMS patients; our analysis, however, demonstrated an increased risk of death for women who are not insured.

Consistent with our findings, retrospective studies in uterine LMS have demonstrated that adjuvant chemotherapy does not have a significant benefit on OS compared to observation in early-stage (stages I-II) uterine LMS patients [8, 16, 17, 20–23]. Our analysis also demonstrated that there was no interaction between stage and chemotherapy; therefore, patients with higher stage disease did not benefit from adjuvant chemotherapy. A multicenter retrospective study of 140 patients with early-stage uterine LMS showed that, after a 5-year follow-up, 68.7% of women treated with chemotherapy were alive compared to 65.6% of patients who were only observed (*P*=0.521) [20]. A total of 37.1% of patients received chemotherapy in this study, and the most commonly used agents were doxorubicin and ifosfamide [20]. The study of Littell et al. in which patients uniformly received gemcitabine and docetaxel as adjuvant therapy saw similar results [8]. Another retrospective study of 167 uterine LMS patients, 70% of whom had nonmetastatic disease (stages I-III), showed that the lack of survival benefit of adjuvant chemotherapy extended to stage III disease [24]. A small (*N*=23) prospective study of gemcitabine-docetaxel in completely resected stage I–IV high-grade uterine LMS included 18 patients with early-stage (I-II) disease. In this group, 59% showed no disease progression at 2 and 3 years [15]. These intriguing data considered to be better than historical controls prompted the phase II adjuvant chemotherapy study in which gemcitabine and docetaxel followed by doxorubicin were administered to patients with resected uterus limited leiomyosarcoma. The results were promising with higher than expected PFS rates at 2 (78%) and 3 (57%) years, respectively [6].

There has been no completed randomized controlled trial testing the hypothesis that chemotherapy may improve outcomes specifically in nonmetastatic uterine LMS. The French Sarcoma Group compared chemotherapy with doxorubicin, ifosfamide, and cisplatin, followed by radiation, to radiation alone in 81 patients with uterine sarcoma [25]. Though the study included high-grade stromal sarcoma and carcinosarcoma, 65.5% of the study population had LMS. There was no improvement in OS in the group receiving chemotherapy followed by radiation; however, there was a statistically significant improvement in 3-year disease-free survival [25]. No comparison to this disease-free survival result can be made from our dataset as the NCDB does not collect information on recurrence. Prior to this study, another randomized controlled trial by Omura et al. studied the effect of adjuvant doxorubicin in all uterine sarcomas [26]. This trial included 156 evaluable uterine sarcoma patients, including 32% stage I or II uterine LMS patients. Patients in the chemotherapy group were given 8 cycles of 60 mg/m^2^ adjuvant doxorubicin every three weeks. There was no statistically significant difference in OS or PFS between the two groups for the entire cohort. However, LMS patients, in particular, showed a recurrence rate of 44% in the doxorubicin arm and 61% percent in the control arm [26]. The only other randomized trial to study the effects of adjuvant chemotherapy on nonmetastatic uterine LMS enrolled only 17.6% of its target accrual [7]. The international phase III trial of gemcitabine and docetaxel followed by doxorubicin was ultimately terminated early due to slow accrual. Though the results of the 38 patients enrolled did not allow for sound statistical analysis, there did not appear to be any significant benefit of chemotherapy on either recurrence-free survival or OS [7].

Given that ours is a retrospective database study, there are some limitations to be acknowledged. The NCDB does not provide specifics on chemotherapy regimens; however, we were able to determine whether patients received single-agent or multi-agent therapy. Many prior studies have focused on the use of gemcitabine and docetaxel or doxorubicin-based chemotherapy. Cases in the NCDB were coded using either AJCC or CS staging, which required us to develop a composite staging system. Therefore, comparison between our results and those of other studies needs to consider the staging system used. Finally, the NCDB reports OS and not disease-specific survival. As in all observational studies, patient selection effects may bias the results; however, we used multiple methods to control for all known confounding variables and demonstrated consistent results.

## 5. Conclusions

Our study demonstrated that expected tumor and patient characteristics such as early stage of tumor and older age were associated with decreased likelihood of receiving chemotherapy. Consistent with the literature, there was no benefit for adjuvant chemotherapy in patients with nonmetastatic uterine LMS. However, we found that the usage of chemotherapy is on the rise. Given the large sample size of our study and the limited preexisting data on the factors impacting chemotherapy in patients with this rare disease, this analysis adds to the existing literature on the practice patterns for chemotherapy use in patients with nonmetastatic uterine LMS.

## Figures and Tables

**Table 1:** Demographic and clinical characteristics of uterine LMS patients stratified by receipt of chemotherapy.

Characteristics	Chemotherapy *N*=936		No chemotherapy *N*=1796		*P*
	Number	%	Number	%	
Age at diagnosis, *y*					**<0.0001**
** **18–39	66	7.1	107	6.0	
** **40–49	270	28.8	467	26.0	
** **50–64	475	50.7	794	44.2	
** **65+	125	13.4	428	23.8	

Race (missing from analysis: *N*=160)					0.7531
** **White	701	79.7	1327	78.4	
** **African American	179	20.3	365	21.6	

Hispanic (missing from analysis: *N*=132)					**0.0292**
** **Yes	79	8.8	111	6.5	
** **No	814	91.2	1596	93.5	

Comorbidities (Charlson/Deyo score)					0.1509
** **0	787	84.1	1460	81.3	
** **1	126	13.5	275	15.3	
** **2	23	2.5	61	3.4	

Insurance status (missing from analysis: *N*=35)					**<0.0001**
** **Medicare	122	13.2	442	24.9	
** **Private	657	71.0	1112	62.8	
** **Medicaid/Other government	89	9.6	120	6.8	
** **None	57	6.2	98	5.5	

Median household income ($) (missing from analysis: *N*=37)					0.4726
** **<38,000	164	17.7	329	18.6	
** **38,000–47,999	198	21.4	366	20.7	
** **48,000–62,999	252	27.2	441	24.9	
** **>63,000	311	33.6	634	35.8	

Education (% no high school diploma) (missing from analysis: *N*=37)					**0.0355**
** **≥21	166	17.9	321	18.1	
** **13–20.9	185	20.0	437	24.7	
** **7–12.9	335	36.2	576	32.5	
** **<7	239	25.8	436	24.6	

Location of patient (missing from analysis: *N*=90)					0.0902
** **Large metropolitan	574	62.7	1013	58.7	
** **Small metropolitan	236	25.8	484	28.0	
** **Suburban	75	8.2	145	8.4	
** **Rural	30	3.3	85	4.9	

Hospital volume (cases per year)					**0.0181**
** **0.09	38	4.1	99	5.5	
** **0.18–0.27	103	11.0	256	14.3	
** **0.36–0.55	181	19.3	353	19.7	
** **0.64–7.27	614	65.6	1088	60.6	

Regional lymph nodes positive (missing from analysis: *N*=1,700)					**<0.0001**
** **Yes	30	8.0	13	2.0	
** **No	347	92.0	642	98.0	

Stage					**<0.0001**
** **1	676	72.2	1541	85.8	
** **2	64	6.8	116	6.5	
** **3	196	20.9	139	7.7	

Year of diagnosis					**<0.0001**
** **2004–2009	345	36.9	1005	56.0	
** **2010–2014	591	63.1	791	44.0	

Radiation (missing from analysis: *N*=21)					**0.0406**
** **Yes	225	24.3	373	20.9	
** **No	700	75.7	1413	79.1	

Oophorectomy (missing from analysis: *N*=184)					0.6492
** **Yes	716	84.0	1437	84.7	
** **No	136	16.0	259	15.3	

**Table 2:** Multivariate predictors of chemotherapy receipt in nonmetastatic uterine LMS.

Variable	OR	95% CI	*P*
Year of diagnosis			
** **Per year increase	1.16	1.13–1.20	**<0.0001**

Age at diagnosis, *y*			
** **18–39	Ref		
** **40–49	0.93	0.67–1.31	0.6927
** **50–64	0.93	0.67–1.29	0.6537
** **65+	0.60	0.38–0.94	0.0264

Race			
** **White	Ref		
** **African American	0.80	0.63–1.02	0.0727
** **Other	1.09	0.78–1.53	0.5965

Hispanic			
** **Yes	1.34	0.94–1.91	0.1112
** **No	Ref		

Comorbidities (Charlson/Deyo score)			
** **0	Ref		
** **1	0.93	0.73–1.18	0.5332
** **2	0.72	0.37–1.41	0.3381

Insurance status			
** **Private	Ref		
** **Medicare	0.59	0.42–0.84	**0.0030**
** **Medicaid	1.06	0.77–1.48	0.7062
** **None	0.92	0.64–1.30	0.6265
** **Other government	2.42	1.04–5.61	**0.0400**

Education (% no high school diploma)			
** **≥21	0.68	0.48–0.97	**0.0354**
** **13–20.9	0.58	0.43–0.79	**0.0005**
** **7–12.9	0.86	0.68–1.09	0.2143
** **<7	Ref		

Median household income ($)			
** **<38,000	1.49	1.04–2.13	**0.0282**
** **38,000–47,999	1.42	1.05–1.92	**0.0239**
** **48,000–62,999	1.37	1.07–1.75	**0.0117**
** **>63,000	Ref		

Location of patient			
** **Large metropolitan	Ref		
** **Small metropolitan	0.92	0.73–1.15	0.4570
** **Suburban	1.00	0.73–1.39	0.9764
** **Rural	0.69	0.43–1.10	0.1146

Hospital volume (cases per year)			
** **0.09	Ref		
** **0.18–0.27	1.00	0.62–1.61	0.9964
** **0.36–0.55	1.18	0.74–1.87	0.4912
** **0.64–7.27	1.27	0.82–1.98	0.2812

Regional lymph nodes positive			
** **Yes	3.83	1.81–8.09	**0.0004**
** **No	Ref		

Stage			
** **I	0.32	0.25–0.41	**<0.0001**
** **II	0.38	0.26–0.56	**<0.0001**
** **III	Ref		

Hormone therapy			
** **Yes	0.72	0.30–1.70	0.4473
** **No	Ref		

Radiation			
** **Yes	1.45	1.17–1.79	**0.0006**
** **No	Ref		

Oophorectomy			
** **Yes	0.92	0.72–1.19	0.5395
** **No	Ref		

## Data Availability

The retrospective data used to support the findings of this study are available in the National Cancer Database.
